# Pancreatic neuroendocrine tumor producing vasopressin: A case report

**DOI:** 10.1097/MD.0000000000027453

**Published:** 2021-10-08

**Authors:** Jingyan Li, Xinxin Zhang, Qing He, Wenli Feng, Li Ding, Zhuoqun Wang, Haonan Yu, Qiusong Chen, Ning Lu, Dongbo Xu, Jingqiu Cui

**Affiliations:** a Department of Endocrinology and Metabolism, Tianjin Medical University General Hospital, Tianjin, China; b Department of Cardiovascular Disease, Tianjin Medical University General Hospital, Tianjin, China; c Department of PET-CT, Tianjin Medical University General Hospital, Tianjin, China; d Department of General Surgery, Tianjin Medical University General Hospital, Tianjin, China; e Department of Pathology, Tianjin Medical University General Hospital, Tianjin, China.

**Keywords:** hyponatremia, pancreatic neuroendocrine tumors, relative adrenal insufficiency, syndrome of inappropriate antidiuresis, vasopressin

## Abstract

**Rationale:**

Functional pancreatic neuroendocrine tumors (pNETs) rarely produce vasopressin. Here, we reported a case of pNET producing vasopressin in a 78-year-old man with hyponatremia.

**Patient concerns:**

The patient presented with anorexia approximately 4 years ago, and the laboratory test results indicated hyponatremia. He was hospitalized 3 times subsequently due to anorexia in the past 4 years, during which laboratory tests consistently indicated severe hyponatremia.

**Diagnosis:**

Upon admission, his serum osmolarity, urine osmolarity, urine sodium level, and 24-hour urine sodium level was 277 mOsm/kg H2O, 465 mOsm/kg H2O, 82.5 mmol/L, and 140.25 mmol, respectively. Gallium-68-labeled tetraazacyclododecanetetraacetic acid-Dphel-Tyr3-octreotate positron emission tomography-computed tomography showed a high uptake lesion measuring approximately 1 cm in diameter in the pancreatic body, and the possibility of pNET was considered. Besides, laboratory tests showed that adrenocorticotropic hormone, follicle-stimulating hormone, and luteinizing hormone released by the pituitary was insufficient in the case of low levels of cortisol, estradiol, progesterone, and testosterone. Thus, the diagnosis of the syndrome of inappropriate antidiuresis (SIAD) was considered along with hypopituitarism.

**Interventions:**

The patient underwent surgery, and pNET was confirmed by pathology examination. The immunohistochemical study showed that the tumor cells were positive for somatostatin receptors 2 and vasopressin.

**Outcomes:**

In the last follow-up 17 months after surgery, the patient was in good condition, taking methylprednisolone 4 mg every other day, and had been free of anorexia or hyponatremia episodes.

**Lessons:**

This case illustrated the potential ectopic production of vasopressin resulting in SIAD in pNETs, highlighting the adoption of gallium-68-labeled tetraazacyclododecanetetraacetic acid-Dphel-Tyr3-octreotate positron emission tomography-computed tomography and vasopressin immunohistochemical staining in the evaluation of the etiology of SIAD.

## 1 Introduction

Pancreatic neuroendocrine tumor (pNET) is a neuroendocrine tumor (NET) that originates from the pancreas. Although pNETs are rare neoplasms, the incidence and prevalence have been steadily increasing.^[1]^ pNETs include both functional pNETs with clinical syndromes caused by substances produced by the tumors and nonfunctional pNETs with no distinct clinical manifestations.^[2]^ Functional pNETs may produce a variety of hormones, including insulin, gastrin, vasoactive intestinal polypeptide, glucagon, somatostatin, growth hormone-releasing hormone, and adrenocorticotropic hormone (ACTH).^[3,4]^

Vasopressin, also known as antidiuretic hormone (ADH), is synthesized in the hypothalamus and stored in the neurohypophysis, which keeps plasma osmolality within a narrow range by regulating the reabsorption of water in the kidneys.^[5]^ It has been hypothesized that an inappropriate release of ADH or an increased renal response to ADH results in the syndrome of inappropriate antidiuresis (SIAD),^[6]^ which manifests as excessive reabsorption of water and dilutional hyponatremia. Conditions that may cause SIAD include: ectopic ADH secretion by malignant tumor cells, for example, small cell lung cancer; increased production of ADH-like substances by the hypothalamus, secondary to trauma, infection, or tumors; infectious lung disease, including tuberculosis, pneumonia, and fungal infection, etc; and secretion of ADH stimulated by drugs, including cytotoxic drugs, anesthetics, and interferons.^[7]^ However, few cases of vasopressin-producing pNETs leading to SIAD have been reported so far.^[8]^

Herein, we reported an unusual case of a pNET ectopically producing vasopressin that resulted in SIAD, which was confirmed by pathological examination and immunohistochemical staining for vasopressin.

## 2 Case presentation

A 78-year-old man was referred to the Department of Endocrinology and Metabolism of our hospital for recurrent episodes of hyponatremia on July 5, 2019. The patient presented with anorexia approximately 4 years ago, and the laboratory test results from the local hospital indicated hyponatremia, which was resolved by symptomatic treatment. The patient was hospitalized 3 times subsequently due to anorexia in the past 4 years, during which laboratory tests consistently indicated severe hyponatremia. The lowest serum sodium level was 121 mmol/L. He was not on any medication known to induce hyponatremia. He did not have a familial history of NET. Upon admission, his blood pressure, pulse rate, respiratory rate, and temperature was 144/74 mm Hg, 60 beats per minute, 20 breaths per minute, and 36.4 °C, respectively. He was alert and had normal skin elasticity. Physical examinations of his heart and lungs were unremarkable. The abdomen was soft, without tenderness, and with normal bowel sounds. There was mild edema in the lower limbs.

**Figure 1:**
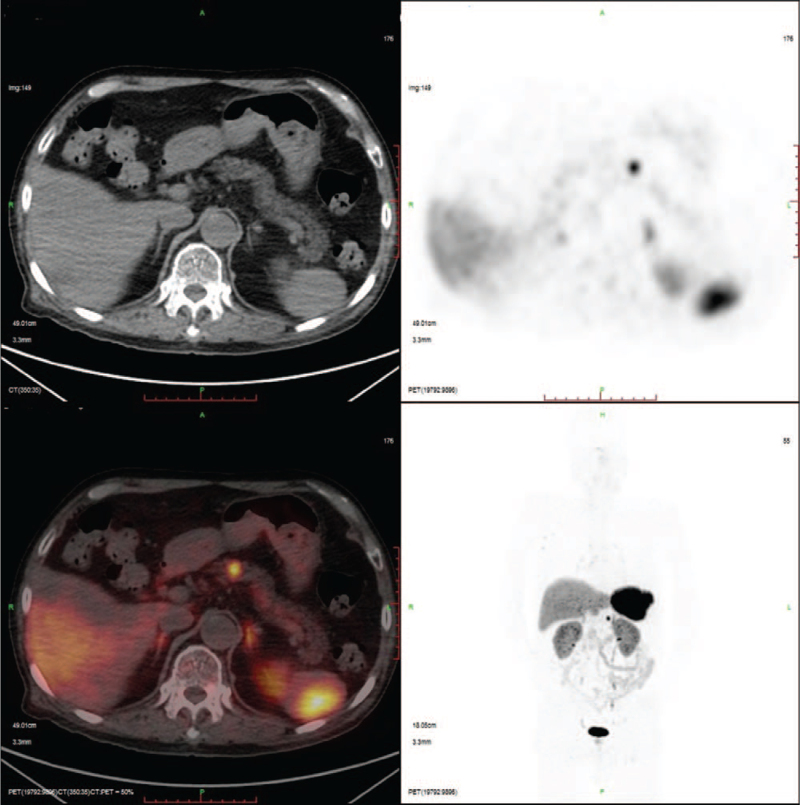
A lesion with high DOTATATE uptake approximately 1 cm in diameter in the pancreatic body showed by the ^68^Ga-DOTATATE PET-CT. ^68^Ga-DOTATATE = Gallium-68-labeled tetraazacyclododecanetetraacetic acid-Dphel-Tyr3-octreotate, PET-CT = Positron emission tomography-CT.

**Figure 2:**
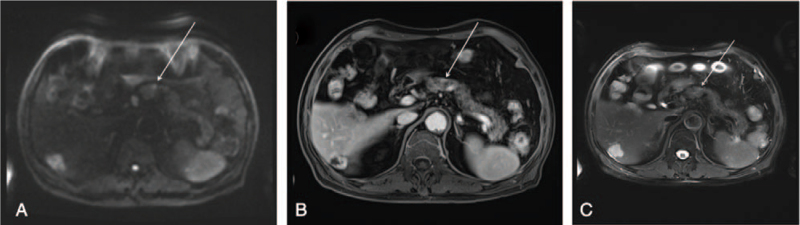
A small nodule (arrows) in the body of the pancreas consistent with the feature of pNET in the non-enhanced and enhanced magnetic resonance (MR) imaging of the upper abdomen. pNET = pancreatic neuroendocrine tumor.

Laboratory findings on admission showed hyponatremia (128 mmol/L; normal, 136-145), glucose 5.5 (normal 3.6-5.8) mmol/L, blood urea nitrogen 1.4 (normal, 2.5-7.1) mmol/L, uric acid 141 (normal, 140-414) μmol/L, and creatinine 58 (normal, 62-133) μmol/L. Serum osmolarity, urine osmolarity, urine sodium, and 24-hour urine sodium was 277 (normal 275-305) mOsm/kg·H_2_O, 465 (normal 600-1000) mOsm/kg·H_2_O, 82.5 mmol/L, and 140.25 (normal 130.00-260.00) mmol/24 hour, respectively. Other laboratory findings are presented in Table 1. The levels of renin and aldosterone were low. Moreover, the ACTH, follicle-stimulating hormone, and luteinizing hormone released by the pituitary were insufficient in the case of low levels of cortisol, estradiol, progesterone, and testosterone. SIAD was suspected along with possible hypopituitarism. The patient underwent positron emission tomography-computed tomography (PET-CT) to exclude the possibility of a tumor. PET-CT using ^18^F-fluorodexyglucose was unremarkable. However, gallium-68-labeled tetraazacyclododecanetetraacetic acid-Dphel-Tyr3-octreotate (^68^Ga-DOTATATE) PET-CT displayed a lesion with high DOTATATE uptake, measuring approximately 1 cm in diameter in the pancreatic body (Fig. 1), which was indicative of the pNET. Furthermore, non-enhanced and enhanced magnetic resonance imaging of the upper abdomen revealed a small nodule in the body of the pancreas, which was consistent with the pNET (Fig. 2). Head magnetic resonance imaging did not show any signs of central nervous system disease. There were no signs and symptoms of heart failure, and left ventricular ejection fraction was normal as assessed by echocardiography. Adrenal computed tomography findings were normal. A diagnosis of SIAD was made on the basis of laboratory and clinical findings. Normal serum sodium levels were maintained for a week after the oral administration of 7.5 mg tolvaptan. Besides, ultrasound of lower extremity vein indicated deep venous thrombosis, which caused pain in the left lower limbs, and diclofenac sodium, a nonsteroidal anti-inflammatory drug, was given.

**Table 1:** Laboratory tests during hospitalization of the patient.

Category	Date	Reference range	Values
TP (g/L)	July 5, 2019	63–82	62
Alb (g/L)	July 5, 2019	35–50	31
Glo (g/L)	July 5, 2019	20–40	31
TC (mmol/L)	July 6, 2019	3.59–5.17	5.23
TG (mmol/L)	July 6, 2019	0.57–1.71	1.76
HDL-C (mmol/L)	July 6, 2019	0.80–2.20	0.52
LDL-C (mmol/L)	July 6, 2019	1.33–3.36	3.95
FT3 (pmol/L)	July 6, 2019	2.63–5.70	2.48
FT4 (pmol/L)	July 6, 2019	9.01–19.05	11.78
TSH (μIU/mL)	July 6, 2019	0.35–4.94	2.914
FSH (IU/L)	July 6, 2019	0.95–11.95	6.34
LH (IU/L)	July 6, 2019	0.57–12.07	3.34
PRL (ng/mL)	July 6, 2019	3.46–19.40	5.87
E2 (pg/mL)	July 6, 2019	11.00–44.00	<10
P (ng/mL)	July 6, 2019	0.00–0.20	<0.10
T (ng/dL)	July 6, 2019	142.39–923.14	19.37
8:00 am cortisol (μg/dL)	July 6, 2019	5.00–25.00	5.71
ACTH (pg/mL)	July 6, 2019	0.00–46.00	27.60
8:00 am cortisol (μg/dL)	July 10, 2019	5.00–25.00	14.90
ACTH (pg/mL)	July 10, 2019	0.00–46.00	33.90
Urinary cortisol (μg/24 h)	July 10, 2019	33.00–110.00	35.02
Renin (recumbent position, uIU/mL)	July 8, 2019	2.8–39.9	1.7
Aldosterone (recumbent, ng/dL)	July 8, 2019	3.0–23.6	<0.97
Renin (recumbent, uIU/mL)	July 15, 2019	2.8–39.9	0.6
Aldosterone (recumbent, ng/dL)	July 15, 2019	3.0–23.6	<0.97
Renin (standing, uIU/mL)	July 17, 2019	4.4–46.1	<0.5
Aldosterone (standing, ng/dL)	July 17, 2019	3.0–35.3	2.1

ACTH = adrenocorticotropic hormone, E2 = estradiol, FSH = follicle-stimulating hormone, FT3 = free triiodothyronine, FT4 = free thyroxine, HDL-C = high density lipoprotein cholesterol, LDL-C = low density lipoprotein cholesterol, LH = luteinizing hormone, P = progesterone, PRL = prolactin, T = testosterone, TC = total cholesterol, TG = triglycerides, TSH = thyroid-stimulating hormone.

**Figure 3:**
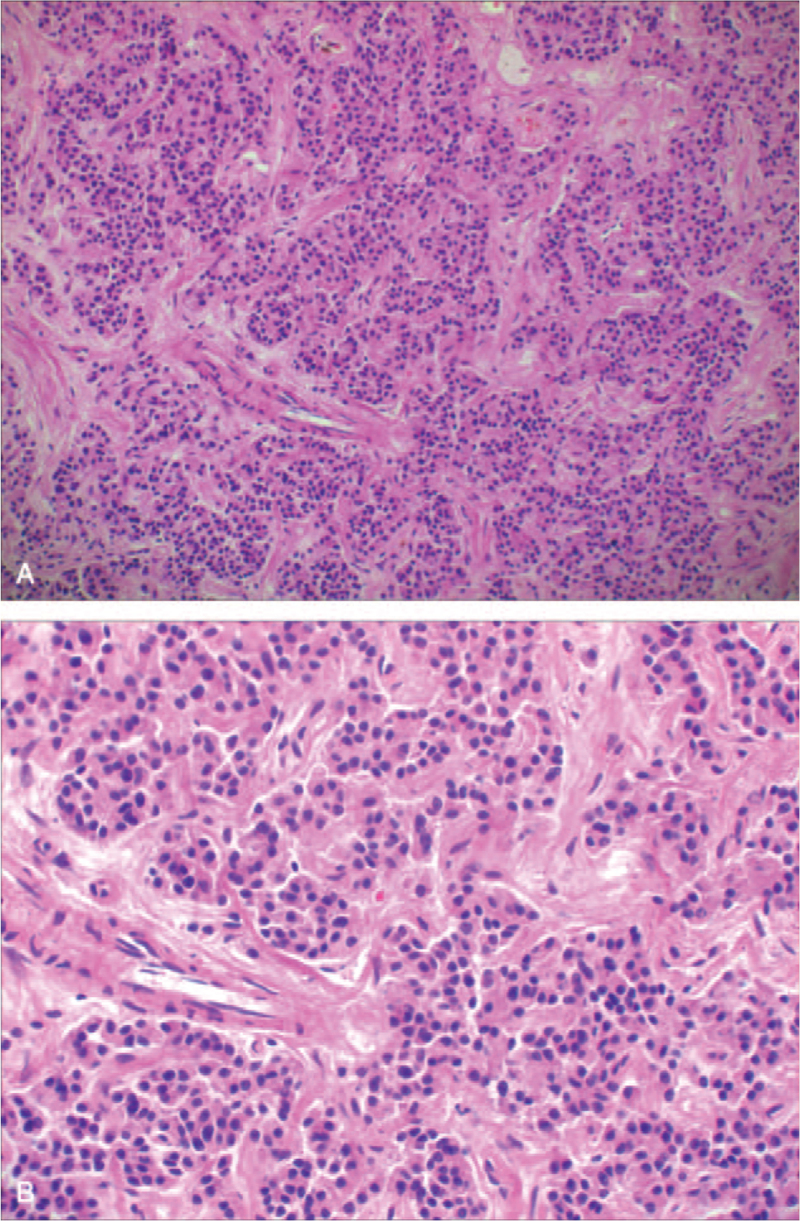
Pathology of the pancreatic neuroendocrine tumor (pNET) (A, H&E stain, ×40; B, H&E stain, ×100). pNET = pancreatic neuroendocrine tumor.

**Figure 4:**
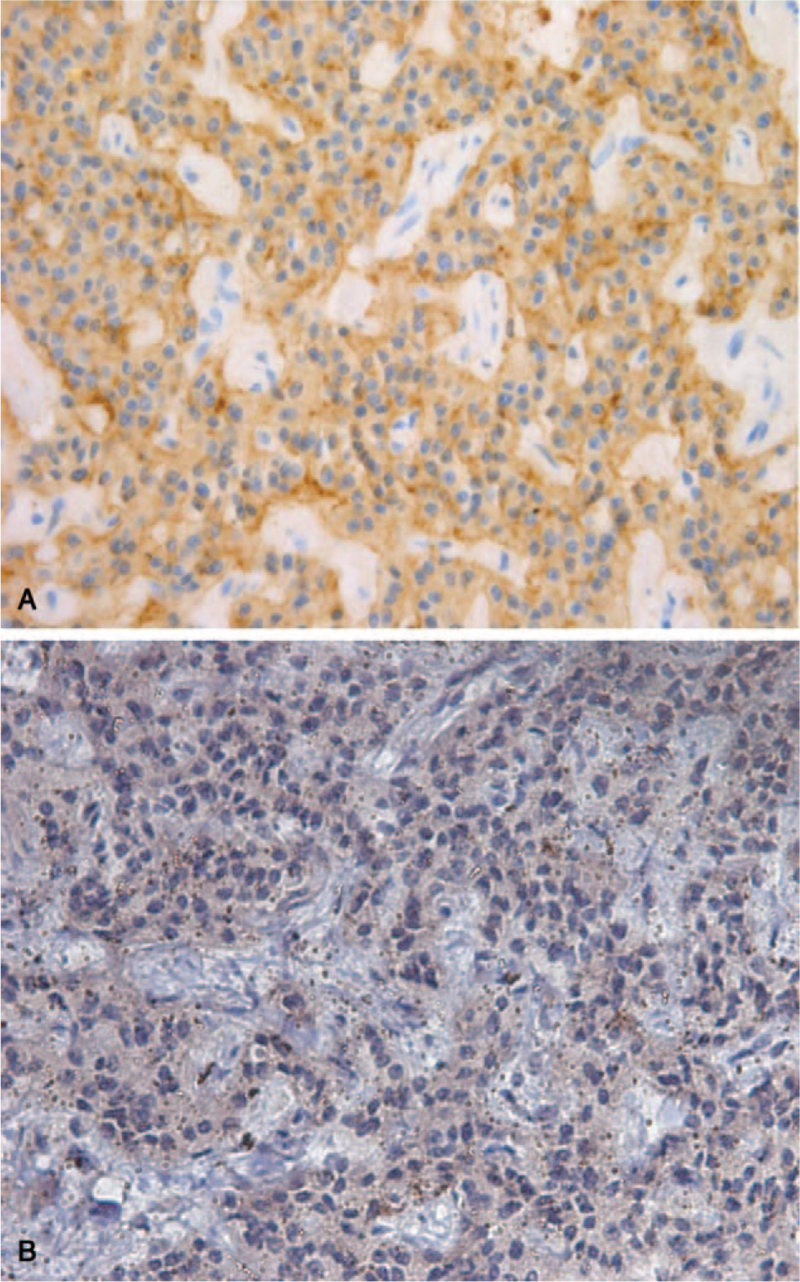
Positivity for SSTR2 (A, immunohistochemical stain, ×40) and vasopressin (B, immunohistochemical stain, ×40) in surgical specimens of the tumor. SSTR2 = somatostatin receptors 2.

The patient underwent laparoscopic-assisted middle segment pancreatectomy and pancreatic-gastric anastomosis on July 31, 2019. A diagnosis of pNETs was confirmed by pathology examination (G2, Fig. 3). Karyokinesis was rare. The Ki-67 index was greater than 3%. Immunohistochemistry showed positivity for CgA, Syn, CD56, CK, somatostatin receptors 2 (SSTR2, Fig. 4A), and vasopressin (NB110-65214, Neurophysin II/Arg-vasopressin Antibody, Novus Biologicals, Fig. 4B) in the tumor cells, indicating a vasopressin-producing pNET.

The patient developed heart failure in the postoperative period. He suffered from hyponatremia again, with a serum sodium level of 134 mmol/L. Because of suspected relative hypopituitarism, intravenous and oral corticosteroids were initiated. The serum sodium level was normalized (142 mmol/L) 5 days after 50 to 100 mg of hydrocortisone treatment daily. The dosage of corticosteroid was gradually tapered. In the last follow-up 17 months after surgery, the patient was in good condition, taking methylprednisolone 4 mg every other day, and had been free of anorexia or hyponatremia episodes. Owing to the outbreak of coronavirus disease 2019 (COVID-19), the patient did not visit our hospital for the recent follow-up.

## 3 Discussion

In this case, the pNET ectopically producing vasopressin was clinically suspected and confirmed in pathology, highlighting the adoption of ^68^Ga-DOTATATE PET-CT and immunohistochemical staining for vasopressin in evaluating the etiology of SIAD.

Hyponatremia is mainly an abnormality of water balance with a relative excess of body water compared to the total sodium content in the body.^[9]^ Sodium ions are the main component of osmotic pressure in the extracellular fluid. The main physiological mechanisms of regulating serum osmolarity are thirst, as well as vasopressin released by the pituitary.^[9]^ Vasopressin is a polypeptide synthesized by the supraventricular nucleus and paraventricular nucleus of the hypothalamus and secreted by the posterior pituitary gland. The main reaction to vasopressin is the increased water reabsorption in the collecting tubules of the kidneys. Hyponatremia is usually related to a disorder of vasopressin that governs water balance.^[9]^

Hyponatremia is the most common electrolyte abnormality observed in clinical practice.^[10]^ The diagnosis of hyponatremia is complex due to its complicated etiology and nonspecific clinical feature. The etiology of hyponatremia is thought to be multifactorial. A prospective study recruiting ambulatory patients indicated approximately half of the patients had a multifactorial etiology of hyponatremia.^[11]^ In our patient, multiple factors contributed to his hyponatremia. Firstly, owing to insufficient ACTH, follicle-stimulating hormone, and luteinizing hormone released by the pituitary in the case of low levels of cortisol, estradiol, progesterone, and testosterone, as well as the effect of methylprednisolone, hypopituitarism was considered in our patient, which could be a possible etiology of his hyponatremia. Secondly, there was vasopressin ectopically produced by pNET in this patient, which was confirmed by pathological examination and immunohistochemical staining for vasopressin. Furthermore, laboratory findings showed that the levels of renin and aldosterone were low. After hospitalization, the patient was prescribed diclofenac sodium to relieve pain in the left lower limbs caused by deep venous thrombosis. It has been demonstrated that the use of nonsteroidal anti-inflammatory drugs will decrease the levels of renin and aldosterone.^[12]^ Thus, the low level of the renin-angiotensin-aldosterone system was an aggravating factor of hyponatremia. Therefore, in this patient, low level of the renin-angiotensin-aldosterone system, hypopituitarism, and the inappropriate production of vasopressin by the pNET jointly resulted in hyponatremia.

Previous studies have indicated that inappropriate ADH secretion may occur as follows: ADH synthesized by the supraventricular nucleus and paraventricular nucleus of the hypothalamus and released by the posterior pituitary gland; ectopic ADH secretion; missense mutation of the V2 vasopressin receptor causing constitutive activation of V2 vasopressin receptor and SIAD-like clinical manifestation of patients.^[13]^ In our patient, we found an inappropriate release of vasopressin by the pNET, which was an unusual cause of SIAD.

As far as we know, there has been only 1 case of vasopressin-producing pNET reported so far. Alshaikh et al^[8]^ reported on a 52-year-old man presenting with intermittent abdominal pain. Core biopsy confirmed a NET originating from the pancreas. The pathology of the NET was positive for pancreatic polypeptide and insulin. At that point, the blood glucose level was 6.5 mmol/L and serum sodium level was 132 mmol/L. Four years later, he developed hypoglycemia accompanied by inappropriately elevated proinsulin and insulin levels. Laboratory findings showed that serum osmolality was 250 mOsm/kg and urine osmolality was 140 mOsm/kg, which were consistent with those of SIAD. The autopsy was diffusely positive for vasopressin, which was not observed in the original biopsy. In this patient, the vasopressin-producing feature of the pNET was confirmed by autopsy when he died of the disease nearly 9 years after the initial diagnosis. This suggests that early diagnosis of the cause of hyponatremia is difficult in clinical practice.

As shown above, the ^68^Ga-DOTATATE PET-CT and immunohistochemistry of vasopressin provided key information in our case. Therefore, it is essential to conduct immunohistochemical staining for vasopressin in patients who could have vasopressin-producing NETs. ^68^Ga-DOTATATE PET-CT is a functional imaging modality used to assess well-differentiated NETs^[14]^ and an effective tool for locating primary tumors in patients with an unknown type of NET.^[15]^ Ga-DOTATATE has the highest affinity for SSTR2, which tends to be most overexpressed in NETs.^[16]^ It has become the preferred imaging method for initial diagnosis, patients inclined to receive peptide receptor radionuclide therapy, and localization of unknown primary tumors.^[14]^ Moreover, a prospective study showed that ^68^Ga-DOTATATE PET-CT changed the treatment of 33 (66%) among 50 patients who underwent this imaging procedure.^[17]^ Thus, for staging and monitoring of NETs, ^68^Ga-DOTATATE PET-CT should be considered as it is usually related to changes in treatment.^[17]^

There are several limitations to our case report. First, we did not measure the ADH level due to the lack of routine analysis of serum ADH in clinical practice. Second, due to the critical condition of our patient and the difficulty in obtaining ACTH, assessment of adrenal function, such as the circadian adrenocortical rhythm and the ACTH stimulation, was insufficient before surgery. Third, we did not receive data on the patient's detailed serum sodium levels after discharge. Due to the influence of COVID-19 on the hospital environments as well as his advanced age, he was unable to visit our department, making follow-up visits difficult. However, we learned that he was in good condition through telephone interviews.

## 4 Conclusion

In conclusion, we reported an unusual case of a pNET ectopically producing vasopressin. The pNET was suggested by ^68^Ga-DOTATATE PET-CT and confirmed by immunohistochemistry of vasopressin. Early diagnosis of the cause of hyponatremia is difficult, so the vasopressin-producing features of pNETs may be undetected in clinical practice. Our case highlights pNETs are possibly 1 of the causes of hyponatremia. A systematic prospective study of SIAD should be conducted to clarify the true prevalence of this phenomenon.

## Author contributions

JL obtained the clinical findings, reviewed the literature, and prepared the figures. XZ wrote the initial manuscript and prepared the figures. QH obtained the clinical findings, reviewed and approved the manuscript. WF performed the immunohistochemical study of SSTR2 and vasopressin. LD reviewed and approved the manuscript. ZW got the patient's history and built the timeline. HY performed the ^18^F-fluorodexyglucose PET-CT and ^68^Ga-DOTATATE PET-CT for the patients. QC was responsible for the diagnosis of the PET-CT. NL operated on the patient. DX made the pathological diagnosis for the patients. JC obtained the clinical findings, reviewed and approved the manuscript, the figures, the diagnosis, and supervised the manuscript.

**Conceptualization:** Jingyan Li, Xinxin Zhang, Qing He, Wenli Feng, Li Ding, Zhuoqun Wang, Haonan Yu, Qiusong Chen, Ning Lu, Dongbo Xu, Jingqiu Cui.

**Data curation:** Jingyan Li, Xinxin Zhang, Qing He, Wenli Feng, Li Ding, Zhuoqun Wang, Haonan Yu, Qiusong Chen, Ning Lu, Dongbo Xu, Jingqiu Cui.

**Formal analysis:** Jingyan Li, Xinxin Zhang, Qing He, Wenli Feng, Li Ding, Zhuoqun Wang, Haonan Yu, Qiusong Chen, Ning Lu, Dongbo Xu, Jingqiu Cui.

**Funding acquisition:** Wenli Feng, Jingqiu Cui.

**Investigation:** Jingyan Li, Xinxin Zhang, Qing He, Wenli Feng, Li Ding, Zhuoqun Wang, Haonan Yu, Qiusong Chen, Ning Lu, Dongbo Xu, Jingqiu Cui.

**Methodology:** Jingyan Li, Xinxin Zhang, Qing He, Wenli Feng, Li Ding, Zhuoqun Wang, Haonan Yu, Qiusong Chen, Ning Lu, Dongbo Xu, Jingqiu Cui.

**Project administration:** Jingyan Li, Xinxin Zhang, Qing He, Wenli Feng, Li Ding, Zhuoqun Wang, Haonan Yu, Qiusong Chen, Ning Lu, Dongbo Xu, Jingqiu Cui.

**Resources:** Jingyan Li, Xinxin Zhang, Qing He, Wenli Feng, Li Ding, Zhuoqun Wang, Haonan Yu, Qiusong Chen, Ning Lu, Dongbo Xu, Jingqiu Cui.

**Supervision:** Jingyan Li, Xinxin Zhang, Qing He, Wenli Feng, Li Ding, Zhuoqun Wang, Haonan Yu, Qiusong Chen, Ning Lu, Dongbo Xu, Jingqiu Cui.

**Validation:** Jingyan Li, Xinxin Zhang, Qing He, Wenli Feng, Li Ding, Zhuoqun Wang, Haonan Yu, Qiusong Chen, Ning Lu, Dongbo Xu, Jingqiu Cui.

**Visualization:** Jingyan Li, Xinxin Zhang, Qing He, Wenli Feng, Li Ding, Zhuoqun Wang, Haonan Yu, Qiusong Chen, Ning Lu, Dongbo Xu, Jingqiu Cui.

**Writing – original draft:** Jingyan Li, Xinxin Zhang.

**Writing – review & editing:** Jingyan Li, Xinxin Zhang, Qing He, Wenli Feng, Li Ding, Zhuoqun Wang, Haonan Yu, Qiusong Chen, Ning Lu, Dongbo Xu, Jingqiu Cui.
